# Values of different specimen preparation methods for the diagnosis of lung cancer by endobronchial ultrasound guided transbronchial needle aspiration

**DOI:** 10.1186/s12890-020-01183-x

**Published:** 2020-05-15

**Authors:** Youzu Xu, Jian Lin, Meifang Chen, Haihong Zheng, Jiaxi Feng

**Affiliations:** 1 Department of Respiratory Medicine, Tai Zhou Hospital of Zhejiang Province, LinHai, Zhejiang, P. R. China; 2 Department of Pathology, Tai Zhou Hospital of Zhejiang Province, LinHai, Zhejiang, P. R. China

**Keywords:** Histopathology, Liquid based cytology, Cytological smear, Endobronchial ultrasound, Lung cancer, Transbronchial needle aspiration

## Abstract

**Background:**

Endobronchial ultrasound guided transbronchial needle aspiration (EBUS-TBNA) has been become an important procedure for the diagnosis and staging of lung cancer. Our research identified the effects of different pathological preparation on the diagnosis of lung cancer for specimens obtained by biopsy.

**Methods:**

Patients were clinically considered if lung cancer was accompanied by mediastinal or hilar lymph node enlargement between March 2014 and November 2017. Specimens obtained by EBUS-TBNA were treated by three methods: traditional smear cytology, liquid-based cytology (LBC) and histopathology.

**Results:**

Of a total of 154 puncture sites from 153 patients, the total positive rate of combination for the three pathological treatment types (histopathology, direct traditional smear, and LBC) was 77.3%. The diagnostic positive rate for histopathology was 68.6%, direct traditional smear was 65.6%, and LBC was 60.4%; there was no significant differences among the three single pathological treatment types (P = 0.29), but there was a statistically significant difference between the combination of three treatments and any single pathological treatment type (P = 0.01). The diagnostic sensitivities of histopathology combined with traditional smear and histopathology combined LBC were 94.4 and 92.8%, respectively, the specificities and PPVs were both 100%, and the diagnostic accuracies were 95.5 and 94.2%, respectively; the sensitivities, specificities and diagnostic accuracies above were all higher than those of single specimen treatment and lower than those of the three combined.

**Conclusion:**

When EBUS-TBNA is used for the diagnosis and staging of lung cancer, the use of histopathological sections combined with direct cytological smear should be sufficient and is the most economical choice.

## Background

In recent years, endobronchial ultrasound guided transbronchial needle aspiration (EBUS-TBNA) has been clinically developed and has achieved positive clinical effects, especially in the diagnosis and staging of lung cancer [1]. As with routine bronchoscopic biopsy, EBUS-TBNA obtains small specimens, and the quality and treatment methods of specimens often affect the final diagnosis of pathology, thus, affecting the clinical diagnosis and treatment of patients. There are different clinical treatment methods for specimens obtained from TBNA, but histopathology, direct cytological smear and liquid-based cytology (LBC) are the main methods used in clinical practice. The results of previous studies of cytology methods are inconsistent. Some studies show that the diagnosis efficiency of direct cytological smear is high, and some show that they are similar; others support the high diagnostic efficiency of LBC [2, 3]. There are also studies that suggest the combination of cytology and histopathology in EBUS-TBNA can significantly improve the diagnostic accuracy of lung cancer. However, the combination of the three methods was unknown, as little previous research data exists [4]. Whether histological specimens are needed after cytological treatment, and whether the two different cytological treatment methods are both necessary is still unknown in clinical practice. How various specimen treatment methods affect the diagnosis, effectiveness and consistency of the results is also unknown. The purpose of this study is to adopt three different treatment methods for EBUS-TBNA specimens to understand their sensitivity, accuracy and consistency in the diagnosis of lung cancer and provide further clinical guidance.

## Methods

### Study subjects

Outpatients and inpatients in our hospital were prospectively screened from March 2014 to November 2017, and they were clinically and radiographically considered for possible lung cancer if presenting with mediastinum or hilar lymph node enlargement and were subjected to EBUS-TBNA. The patients were generally in good condition, without severe cardiac and pulmonary insufficiency and coagulation disorders. All patients were over 18 years old. Prior to the examination, the purpose, methods, procedures of puncture, possible complications and treatment measures were introduced in detail to the patients, and the patients signed a written informed consent. This study was approved by the Clinical Trial Ethics Committee in Taizhou hospital of Zhejiang Province.

### EBUS-TBNA puncture

Patients had no contraindication for bronchoscopy, the puncture was performed in the bronchoscopy examination room of the outpatient department. The patients fasted before the operation. A total of 2% lidocaine was used to achieve anaesthesia by thyrocricoid puncture, and 2 mg of midazolam was used intravenously for sedation. First, routine bronchoscopy was performed and then BF-UC260F-OL8 (Olympus Ltd. Tokyo) was used to enter the airway through the mouth to the check and the target lymph nodes and surrounding blood vessels. The diameter of the target lymph nodes was recorded by the ultrasonic image processing device, a 22G special puncture needle was used to enter the lesion with the penetration method, and the negative pressure syringe was connected to the end of the biopsy needle. Each lymph node was punctured 3 times; if the lump was near the trachea, it could also be punctured, and the method is described above.

### Specimen preparation procedure

#### Traditional direct smear method production cytology

The tissue obtained by the every needle punctures was pushed onto the plain glass with the needle core, and the excess tissue was then clamped by papers into the vial containing formalin. The tissue on glass slide was made into two cytology specimens by the direct smear method, fixed with 95% ethanol for 15 min, stained with HE, and observed under a light microscope.

#### Liquid thin-layer cytology technology production

The tissue obtained by the needle puncture was pushed onto the plain glass with the needle core, then rinsed the residue of the needle with physiological saline into the liquid base testing bottle for the test. The sample was centrifuged at a radius of 10 cm at 1500 r/min for 5 min and the supernatant was discarded; 25 ml of cleaning fluid was added and then oscillated prior to centrifugation at 1500 r/min for 5 min; the supernatant was discarded again, and the sediment was transferred into a Thinprep liquid then oscillated and mixed. After 15 min, an ultrathin cell smear was made by a TCT microcomputer processing system, fixed with 95% ethanol for 15 min, stained with HE, sealed, and observed under a light microscope. If necessary, cell blocks were made.

#### Histopathological examination

The tissue obtained by the needle punctures was coated on the slide, and the visible tissue was put into a small bottle containing 10% formalin for examination. After treatment, the samples were subjected to paraffin embedding, 5 μm-thick continuous sectioning, HE staining, microscopic examination, and immunohistochemical examination when necessary.

**Fig. 1:**
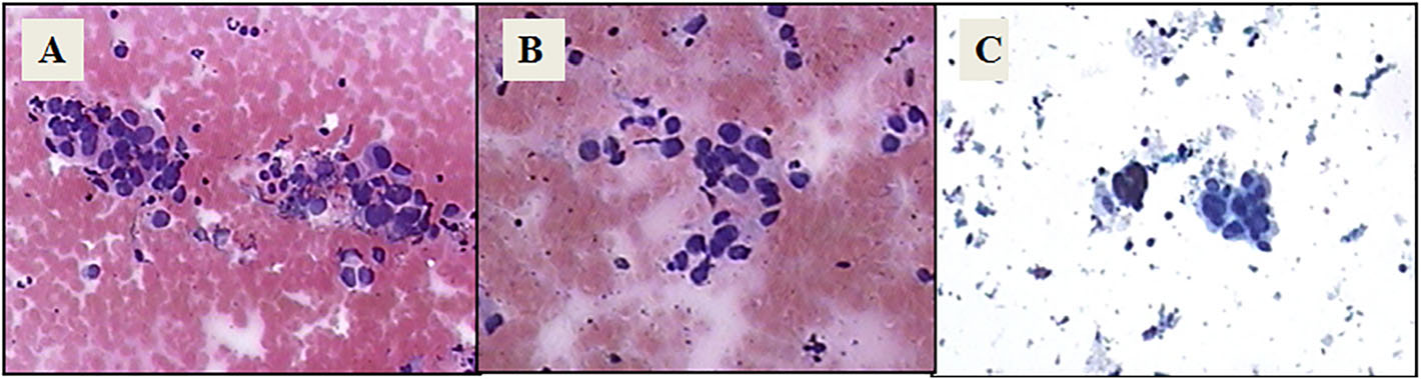
Pathological pictures of three kinds of specimen preparation. **a**: Histology image of lung cancer, HE stain; **b**: Cytology image of lung cancer, HE stain; **c**: liquid-based cytology image of lung cancer, papanicolaou stain. Magnification×100

Specimens from each of the three methods were diagnosed by three different pathologists, and the pathologists did not communicate with one another. The sensitivity and diagnostic accuracy of the three kinds of treatment methods were compared by analysing the treatment methods, pathological results and final disease diagnosis. The three methods were compared to diagnose the consistency. Pathological pictures of three kinds of specimen preparation as shown in Fig. 1.

#### Definitions of diagnostic standards

In our study, EBUS-TBNA results were considered positive when definite malignant tumour cells were detected by cell or histopathological examination. Pathologic findings of highly suspicious malignant cells and clinical manifestations of highly suspected lung cancer or other histologic or cytologic examination proving lung cancer were also considered positive for EBUS-TBNA results and final diagnosis. The pathological results suggest that patients with nonspecific lymphadenopathy who were considered normal or could not be diagnosed and needed further examination, including possible surgical operations (such as thoracoscopy, thoracotomy, mediastinoscope) and CT guided puncture biopsy. If no definite diagnosis was found after all examinations, at least 6 months of clinical follow-up was required.

#### Data processing and statistical analysis

The clinical and pathological characteristics of patients were recorded and collated. The counting data were analysed by χ2 test, and the measurement data were represented by (x ± s) and analysed by a T-test. SPSS 20.0 statistical software was used for the analyses, and two-bilateral p < 0.05 was statistically significant.

## Results

### Patient characteristics

A total of 160 patients were initially screened, of which, 2 patients had only 1 type of pathological specimen and 5 patients had only 2 types of pathological specimens; so these 7 patients were eliminated. Ultimately, a total of 153 patients were included in this retrospective study. Among the study group, 122 cases were male, 31 cases were female, and the average age was 63.2 ± 9.2 (41–81). A total of 154 lymph nodes and tumour puncture samplings were analysed, of which, 1 patient had 2 groups of mediastinal lymph node puncture samples. Specific mediastinal lymph nodes and tumour cases are shown in Table 1. A total of 119 puncture sites were directly diagnosed as lung cancer by EBUS-TBNA. Only 1 patient in the mediastinal 4R and 7 group of lymph nodes had lung cancer; 118 cases were directly diagnosed as lung cancer, including adenocarcinoma in 34 cases, squamous cell carcinoma in 29 cases, non-small cell carcinoma in 19 cases, poorly differentiated carcinoma in 3 cases, small cell carcinoma in 22 cases, and unable to divide cancer cell types in 11 cases.

**Table 1:** Patient characteristics and mediastinal lymph node characteristics

Characteristic	Value
Number of patients	153
Age, median (range) in years	63.2(41–81)
Sex	
Females	31
Males	122
Number of Lymph nodes	154
Lymph node size, median (cm)	1.86 ± 0.60
Lymph node station	
2R	2
4R	53
4 L	11
7	55
10R	5
10 L	2
11R	5
11 L	5
12R	2
Mass	14

### Comparison of positive rates of different pathological diagnosis

In the 154 puncture sites, the total positive rate of combination of three pathological treatment types (histopathology, direct traditional smear, liquid-based cytology) was 77.3% (119/154). The diagnosis of positive rates of single pathological treatment were: tissue pathology 68.6% (106/154), direct traditional smear 65.6% (101/154), and liquid base cytology 60.4% (93/154). There was no significant difference among the three single pathological treatment types (p = 0.29), but there was a statistically significant difference between the combination of the three treatment types and any single pathological treatment type (p = 0.01).

The comparison of positive diagnostic rates of different mediastinal lymph node puncture sites is shown in Table 2. Of the 66 punctures near the trachea mediastinal lymph nodes (including 2R, 4R, 4 L), the total positive rate of combination of the three types of pathological treatments (histopathologic, traditional direct smear, liquid based cytology) was 87.9% (58/66). The diagnostic positive rates of single pathologic treatment type were as follows: histopathology was 78.8% (52/66), traditional smear was 75.8% (50/66), liquid-based cytology was 71.2% (47/66), and there was no statistically significant difference between the three pathological treatment types (P = 0.60). In addition, there was no statistically significant between the combination of three treatment types and any single pathological treatment type (P = 0.11).

**Table 2:** Comparison of positive rates between three different treatment methods of specimens from different puncture sites

Variables	Number	% Total Positive	histopathology % Positive	TS % Positive	LBC % Positive	*P* Value
ALL	154	77.3(119)	68.8(106)	65.6(101)	60.4(93)	0.29^a^, 0.01^b^
Anatomic site						
Trachea mediastinal						
lymph nodes						
(2R + 4R + 4 L)	66	87.9(58)	78.8(52)	75.8(50)	71.2(47)	0.60^a^, 0.11^b^
7 group	55	72.7(40)	65.5(36)	60.0(33)	54.5(30)	0.51^a^, 0.23^b^
Hilar lymph node						
(10R,L + 11R,L + 12R)	19	42.1(8)	31.6(6)	31.6 (6)	26.3(5)	0.92^a^, 0.77^b^
Mass	14	92.9(13)	85.7(12)	85.7(12)	78.6(11)	0.84^a^, 0.75^b^

*TS* Traditional smears, *LBC* liquid-based cytology

^a^: Comparison of positive rates among three single pathological treatment type

^b^: Comparison of positive rates between combination of the three treatment types and any single pathological treatment type

For 55 punctures for 7 groups (Subcarina) of mediastinal lymph nodes, the total positive rate of combination of three pathological treatment types (histopathology, direct traditional smear, liquid based cytology) is 72.7% (40/55). The diagnostic positive rate for single types of pathological treatment were as follows: histopathology 65.5% (36/55), direct traditional smear 60.0% (33/55), and liquid-based cytology 54.5% (30/55); there were no statistically significant differences (p = 0.60) between three single pathological treatment types, and there was no statistically significant correlation (p = 0.11) between the combination of three treatment types and any single pathological treatment type.

Of 19 punctures in pulmonary hilar lymph node (including 10R, 10 L, 11R, 11 L, 12R), the total positive rate of combination of three pathological treatment types (histopathology, direct traditional smear, liquid based cytology) was 42.1% (8/19), and the diagnostic positive rates for single types of pathological treatment were as follows: histopathology 31.6% (6/19), direct traditional smear 31.6% (6/19), liquid-based cytology 26.3% (5/19); there were no statistically significant differences between three single pathological treatment types (P = 0.92) and there was no statistically significant correlation between the combination of three treatment types and any single pathological treatment type (P = 0.77).

Of 14 punctures in paratracheal mass, the total positive rate of combination of three pathological treatment types (histopathology, direct traditional smear, liquid based cytology) was 92.9% (13/14). The diagnostic positive rates for the single type of pathological treatment were as follows: histopathology 85.7% (12/14), direct traditional smear 85.7% (12/14), liquid-based cytology 78.6% (11/14); there were no statistically significant differences between three single pathological treatment types (P = 0.84), and there was no statistically significant correlation between the combination of three treatment types and any single pathological treatment type (P = 0.75).

### Diagnostic efficiency of different specimen treatments

The sensitivity, specificity, positive predictive value, negative predictive value and diagnostic accuracy of a single pathological type for lung cancer diagnosis were as follows: histopathology was 90.6, 100, 100, 77.1, and 92.9%, respectively, direct traditional smear was 84.9, 94.3, 98.1, 64.7, and 87.0%, respectively, and liquid-based cytology was 80.2, 94.7, 97.9, 61.0, and 83.8%, respectively. Comparison of diagnostic accuracy among three single pathological treatment types, there was statistically difference (p = 0.04), the diagnostic accuracy of histopathology is the best.

The sensitivity, specificity, positive predictive value, negative predictive value and diagnostic accuracy of the combination of two pathological types for lung cancer diagnosis were as follows: when histopathology combined with direct traditional smear, they were 94.4, 100, 100, 81.1 and 95.5%, respectively; when histopathology combined with liquid-based cytology, they were 92.8, 100, 100, 76.3, and 94.2%, respectively.

The sensitivity, specificity, positive predictive value, negative predictive value and diagnostic accuracy of the three pathological treatment types combined for lung cancer diagnosis were 96.7, 100, 100, 88.6 and 97.4%, respectively. Comparison of diagnostic accuracy among combination of the three treatment types and combination of the any two treatment types, there was no statistically difference (p = 0.62). as shown in Table 3.

**Table 3:** Comparison of diagnostic values between LBC, Traditional smear and Histopathology

Variables	Sensitivity	Specificity	PPV	NPV	DA	*P* Value
HP, %	90.6%	100%	100%	77.1%	92.9%	
TS, %	84.9%	94.3%	98.1%	64.7%	87.0%	
LBC, %	80.2%	94.7%	97.9%	61.0%	83.8%	0.04^a^
HP + TS, %	94.4%	100%	100%	81.1%	95.5%	
HP + LBC, %	92.8%	100%	100%	76.3%	94.2%	
HP + TS + LBC, %	96.7%	100%	100%	88.6%	97.4%	0.62^b^

*HP* Histopathology, *TS* Traditional smears, *LBC* liquid-based cytology, *PPV* positive predictive value, *NPV* negative predictive value, *DA* diagnostic accuracy

^a^: Comparison of diagnostic accuracy among three single pathological treatment type

^b^: Comparison of diagnostic accuracy among combination of the three treatment types and combination of the two treatment types

### Diagnostic consistency of the three pathological treatments

**Fig. 2:**
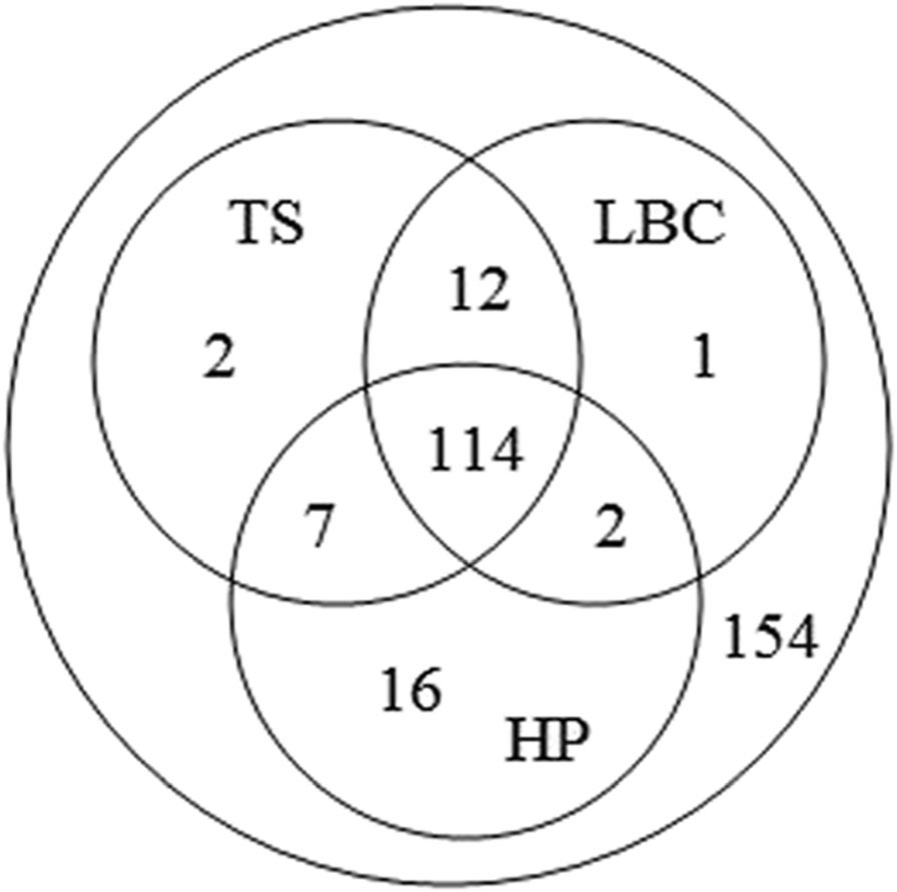
Comparison of diagnostic consistency of three pathological treatment methods. (TS: traditional smears, LBC: liquid-based cytology, HP: histopathology)

Of the 154 puncture sites, the same diagnosis (all positive or negative) were obtained by three methods in 114 cases, accounting for 74.0%. The positive results of traditional direct smear and liquid based cytology were the same, but they do not agree with the result of histopathology in 12 cases, which accounted for 7.7%. Only histopathology and the traditional direct smear had the same positive results in 7 cases, accounting for 4.5%. Only histopathology and liquid based cytology has the same positive diagnosis in 2 cases, which accounted for 1.3%. Only histopathology had single positive results in 16 cases, accounting for 10.4%. There were only 2 cases with single positive results of direct traditional smear, accounting for 1.3%. Only 1 case with single positive diagnosis of liquid-based cytology accounted for 0.6%, as shown in Fig. 2.

## Discussion

In recent years, the incidence and mortality of lung cancer continues to increase. EBUS-TBNA plays an increasingly important role in the diagnosis and staging of lung cancer, due to being minimally invasive and easy to operate. There are different clinical treatments for specimens obtained by TBNA, such as histopathology, direct smear, liquid method, liquid-based cell method, etc. Wang et al. [5] proposed in an earlier paper that needle aspiration specimens should be injected into the container, sent to the laboratory as liquid suspension, and then further examined; this is called the liquid method. Similarly, specimens can also be placed directly on the slide, immediately smeared and spray fixed; this is called the direct method. Direct cytological smear has characteristics of being simple and easy to operate with a rapid diagnosis and high accuracy, but it is easily affected by many factors, such as the production method. Andreas H, Diacon et al. showed in prospective control studies that the positive rate of specimens from direct cytological smear is higher than that from the liquid method, and they were 36.2 and 12.4% respectively (P < 0.01) [6]. Liquid-based cytology (LBC) is a new method of cytology production that has emerged in recent decades. It has been widely used in cervical specimens and has achieved significant effects. It can remove the influence of blood and mucous fluid, making the background of smear clean and the cell structures more clear [7, 8]. but is seldom used in TBNA samples. Gang Hou et al. [9] found that the sensitivity of TBNA samples was 64.7% (65/102) by the traditional direct smear method, while the sensitivity by LBC was 59.8% (61/102). There was no difference between the two methods (P > 0.05), and the use of a combination of direct smear and LBC did not significantly improve the sensitivity of diagnosis. Lee et al. [10] reported that the sensitivity and accuracy of diagnosis of LBC used alone for Endoscopic ultrasonic-guided Fine Needle Aspiration (EU-FNA) was 75.0 and 81%, respectively. Gauchotte et al. [4] reported that the sensitivities of LBC and direct smear were 62.1 and 75.0%, respectively. LBC alone has no advantages, but the sensitivity of combined application with direct smear and needle core biopsy is 96.3%, which was obviously improved.

Specimens of patients with clinically highly suspicious lung cancer obtained by EBUS-TBNA, were treated by three different methods (histopathology, direct cytological smear and LBC method). Through data analysis, we found that the positive rate of the combination of histopathology, direct cytological smear and LBC method was 77.3%, which was significantly higher than that of any single method (P = 0.01). Although there was no difference in the positive rate of specimen treatment results according to different puncture stratification comparisons at different sites (p > 0.05), it was found that the sensitivity and diagnostic accuracy of combined use of the three treatments were also significantly higher than that of the single method or two types combined. Three different specimen treatment was used alone to diagnose, the sensitivity, and positive rate and the diagnostic accuracy of histopathologic was the highest, followed by direct smear, and the lowest by LBC. The diagnostic sensitivities of histopathology, whether combined with traditional images or with LBC, reached more than 90%, and the specificities and PPVs were 100% respectively; all were lower than those of the combination for the three specimen treatments, but there was no statistically significant difference (P > 0.05). The above results indicate that the clinical specimen obtained by EBUS-TBNA for the diagnosis and staging of lung cancer may be almost sufficient for the combined use of two specimen treatments. In terms of benefit economics, the cost of ordinary smear in our hospital is RMB 39 yuan, while the cost of LBC is RMB 155 yuan. Although the accuracy rate of histopathological diagnosis has reached 92.9%, but the traditional smear is more economical and convenient, it plays a role in rapid diagnosis. However, in a small number of suspected cases of use of combination of histopathology and traditional smear, a definite diagnosis can be obtained through LBC. Thus, LBC played a beneficial supplement for the use of a combination of histopathologic and direct smears together, which could improve final diagnostic accuracy and sensitivity.

A limitation of this study was that the endoscopic operator cannot be blinded. This was also a single research unit, not a multi-centre study and that may have inherent research bias; we also did not carry out rapid on-site cytological evaluations due to shortage of manpower and funds.

## Conclusion

When EBUS-TBNA was used to diagnose patients who may have mediastinum or hilar lymph node enlargement with lung cancer, a combination of histopathological slices and traditional direct cytological smear should be sufficient and are the most economical.

## Data Availability

The datasets used and/or analyzed during the current study are available from the corresponding author on reasonable request.

## Abbreviations

EBUS-TBNA: Endobronchial ultrasound guided transbronchial needle aspiration
LBC: Liquid-based cytology
TCT: Thinprep cytologic test
HE: Hematoxylin-eosin
EU-FNA: Endoscopic ultrasonic-guided Fine Needle Aspiration
TBNA: Transbronchial needle aspiration
TS: Traditional smears
HP: Histopathology
